# Glycolysis inhibition via mTOR suppression is a key step in cardamonin-induced autophagy in SKOV3 cells

**DOI:** 10.1186/s12906-018-2380-9

**Published:** 2018-12-04

**Authors:** Daohua Shi, Di Zhao, Peiguang Niu, Yanting Zhu, Jintuo Zhou, Huajiao Chen

**Affiliations:** Department of Pharmacy, Fujian Provincial Maternity and Children’s Hospital, Affiliated Hospital of Fujian Medical University, 18 Daoshan Road, Fuzhou, 350001 Fujian China

**Keywords:** AMPK, Autophagy, Cardamonin, Glycolysis, mTOR

## Abstract

**Background:**

Autophagy occurs in cells that undergoing nutrient deprivation. Glycolysis rapidly supplies energy for the proliferation of cancer cells. Cardamonin inhibits proliferation and enhances autophagy by mTORC1 suppression in ovarian cancer cells. Here, we investigate the relationship between cardamonin-triggered autophagy and glycolysis inhibition via mTORC1 suppression.

**Methods:**

Treated with indicated compounds, ATP content and the activity of hexokinase (HK) and lactate dehydrogenase (LDH) were analyzed by the assay kits. Autophagy was detected by monodansylcadaverin (MDC) staining. The relationship between cardamonin-triggered autophagy and glycolysis inhibition via mTORC1 suppression was analyzed by Western blot.

**Results:**

We found that cardamonin inhibited the lactate secretion, ATP production, and the activity of HK and LDH. The results demonstrated that cardamonin enhanced autophagy in SKOV3 cells, as indicated by acidic compartments accumulation, microtubule-associated protein 1 Light Chain 3-II (LC3-II) and lysosome associated membrane protein 1 up-regulation. Our results showed that the activation of mTORC1 signaling and the expression HK2 were reduced by cardamonin; whereas the phosphorylation of AMPK (AMP-activated protein kinase) was increased. We also confirmed that the AMPK inhibitor, Compound C, reversed cardamonin-induced upregulation of LC3-II.

**Conclusion:**

These results suggest that cardamonin-induced autophagy is associated with inhibition on glycolysis by down-regulating the activity of mTORC1 in ovarian cancer cells.

## Background

Autophagy is a catabolic membrane-trafficking process that mediates the degradation of intracellular material within lysosomes. The cytoplasmic contents are degraded into free nucleotides, amino acids and fatty acids, which are reused by the cell to maintain macromolecular synthesis and to fuel energy production [1]. Autophagy serves as either tumor suppressor or protector in different conditions. It degrades damaged mitochondria and other cell organelles to maintain cell survival. However, excessive autophagy leads to progressive consumption of cellular components, and ultimately induces cell death [2, 3]. Increasing evidence indicates that induction of autophagy inhibits tumorigenesis and cancer progression, especially in certain ovarian cancers which exhibits lack of autophagy [4].

Autophagy is an evolutionarily conserved mechanism of adaptation to microenvironmental conditions, such as metabolism inhibition. It ensures the survival and proliferation of cancer cells under hypoxia and energy starvation [5]. Several signaling pathways capacitate cancer cells to acquire and metabolize nutrients in a manner conducive to proliferation rather than efficient ATP production. Cancer cells produce energy via the conversion of glucose into lactate, despite the presence of oxygen, a process known as aerobic glycolysis. It generates the precursors needed for the synthesis of lipids and nucleotides [6]. Therefore, glycolysis is another evolutionarily mechanism which provides materials for growth and proliferation of cancer cells. Moreover, production of lactic acid can stimulate the invasion and metastasis of cancer cells [7]. Here, we postulate that glycolysis inhibition results in energy deprivation and further triggers autophagy, which ultimately leads to autophagic cell death.

The mammalian target of rapamycin (mTOR) is a key regulator of protein synthesis and cell proliferation. It considered to be an appealing therapeutic target. Makinoshima et al. indicates that mTORC1 regulates glucose uptake, glycolysis and lipid biosynthesis in cancer cells [8]. And mTOR inhibition contributes to the initiation of autophagy even in the presence of sufficient nutrients and growth hormones. Inhibition on the mTOR/S6K1 pathway triggered caspase-dependent and -independent cell death in lymphoma cells by inhibition of glycolysis and induction of autophagy [9, 10].

Cardamonin (*2′, 4′-dihydroxy-6′-methoxy-chalcone*) is the main chalcone found in large amounts of cardamom spice (fruits of *Amomum subulatum* Roxb.) and other medicinal plants of Zingiberaceae family, such as *Alpinia katsumadae* Hayata [11]. Cardamonin appears to have anti-inflammatory, antineoplastic, vasorelaxant and anti-infectious properties which benefit for the potential health applications [12]. Cardamonin inhibits the proliferation and metastasis of various cancer cells [13–15]. In our previous studies, we demonstrate that the antitumor effect of cardamonin dependents on mTOR inhibition [16, 17]. Recently, we find that the autophagy induction is account for the antiproliferative activity of cardamonin [18]. Also, cardamonin plays an important role in glucose metabolism. It eliminates the negative feedback of mTOR/S6K1 on the insulin-signaling pathway and increases glucose metabolism in insulin resistant vascular smooth muscular cells [14, 17, 19].

In the present study, we aim to explore the mechanism of cardamonin on autophagy. The relationship between cardamonin-triggered autophagy and glycolysis inhibition via mTORC1 suppression is investigated in SKOV3 cells. It will provide a promising therapeutic agent for ovarian cancer.

## Methods

### Cells and reagents

Human ovarian cancer SKOV3 cells were obtained from Wuhan boster biological engineering co., LTD (Wuhan, China). Cardamonin, rapamycin, 2-deoxy-D-glucose (2-DG) and Compound C (an AMP-activated protein kinase (AMPK) inhibitor) were purchased from Sigma-Aldrich Chemical Co (St. Louis, MO, USA). The assay kits of ATP, lactate, hexokinase (HK) and lactate dehydrogenase (LDH) were purchased from Nanjing Jiancheng Bioengineering Institute (Nanjing, China). The BCA protein assay kit and antibodies for mTOR, p-mTOR (Ser2448), S6K1, p-S6K1 (Thr389), AMPK, p-AMPK (Ser172), LC3, LAMP1, HK2 and actin were obtained from Cell Signaling Technology (Beverly, MA, USA).

### Cell culture

Human ovarian cancer SKOV3 cells were grown at 37 °C in McCoy’s 5A supplemented with 10% fetal bovine serum (Grand Island, NY, USA), 100 U·mL^− 1^ penicillin and 100 μg·mL^− 1^ streptomycin in a humidified 5% CO_2_ atmosphere.

### Measurement of lactate

Cells were implanted at a density of 2 × 10^5^ cells/well in 6 well plates and cultured for 24 h, then the cells were treated with cardamonin (5, 10, 20 μM), rapamycin (0.1 μM), and 2-DG (10 mM) for another 12, 24, 36, 48, 60 h, respectively. The media was collected and centrifugated (8000 *g*, 10 min) for lactate assay according to the manufacturer’s protocol of Lactate Assay Kits, respectively. The content of lactate was normalized to cell numbers. The inhibitory effect of cardamonin on lactate secretion at 24 h is similar to 12 h in some groups. Compared with 12 and 24 h, the inhibitory effect of cardamonin is significantly increased at 36, 48 and 60 h, respectively. Therefore, we chose the 24 h for the statistical analysis.

We have investigated the antiproliferation effect of cardamonin in various cancer cells. It demonstrated that the efficiency-concentration ranged from 5 μM to 30 μM [13, 15, 16]. In the present study, the inhibitory effect of cardamonin (5, 10 and 20 μM) on the lactate secretion was measured in SKOV3 cells. In order to investigate the further mechanism of cardamonin on glycolysis, we indicated 5, 20 μM or 20 μM of cardamonin for the following experiments.

### Determination of content of ATP and activity of HK and LDH

Treated with indicated drugs as described above, the cells were washed twice with ice cold PBS and harvested with 0.25% trypsogen. The cell suspension was washed with ice cold PBS for three times. Then 300 μL hot distilled water was added to the cells and the mixture was placed in a hot water bath (90~100 °C) for homogenized broken following a boiling water bath for 10 min. At last the ATP was extract from the mixture for 1 min. The ATP absorbance values were measured with the multi-plate reader at 636 nm. Then the cells were crushed with homogenate in ice cold water for HK and LDH activity assay, followed by centrifugation (10,000 *g*, 10 min). The protein content was measured by the BCA assay kit. The ATP content and HK, LDH activity were normalized to total protein. The prepared reagent was pre-warmed at 37 °C for 10 min, and then 50 μL of liquid sample and 960 μL of reagent were immediately mixed in a tube to start the reaction. The absorbance at 340 nm (optical path: 0.5 cm) was recorded after 30 s (OD1) using a Spectrophotometer. Subsequently, the liquid was transferred back to the previous tube and warmed in a 37 °C water bath for 2 min. The absorbance was measured again under the same conditions and denoted as OD2. The HK activity was calculated by the following formula according to manufacturer’s instructions:

$$ \mathrm{HK}\ \mathrm{activity}\ \left(\mathrm{U}/\mathrm{gprot}\right)=\left(\mathrm{OD}2-\mathrm{OD}1\right)/6.22\times \left(1.01/0.05\right)\div \mathrm{C}\ \left(\mathrm{protein}\ \mathrm{concentration}\right) $$

50 μL of liquid sample and 300 μL of reagent were immediately mixed in a tube to start the reaction. The tube was cultured at 37 °C water bath for 15 min. And then another reagent was added in the same tube and cultured at 37 °C water bath for 15 min. At last 2.5 mL 0.4 M NaOH solution was added in the mixture and cultured at room temperature for 3 min. The absorbance at 440 nm (optical path: 1 cm) was recorded using a Spectrophotometer. The LDH activity was calculated by the following formula according to manufacturer’s instructions:

$$ \mathrm{LDH}\ \mathrm{activity}\ \left(\mathrm{U}/\mathrm{gprot}\right)=\left({\mathrm{OD}}_{\mathrm{sample}}-{\mathrm{OD}}_{\mathrm{control}}\right)/\left({\mathrm{OD}}_{\mathrm{standard}}-{\mathrm{OD}}_{\mathrm{control}}\right)\times 2\ \mathrm{mM}\div \mathrm{C}\ \left(\mathrm{protein}\ \mathrm{concentration}\right) $$

### Detection of autophagosome

Cells were cultured in 6-well plates (0.3 × 10^6^/well) for 24 h and then treated with cardamonin (5, 20 μM), rapamycin (0.1 μM), and 2-DG (10 mM) for another 48 h. Cells were washed with ice cold PBS twice and then incubated with monodansylcadaverin (MDC, 0.05 mM) at room temperature in darkness for 30 min. Then the cells were washed twice with PBS and observed by fluorescence microscope. The fluorescent intensity of MDC stain was calculated by the *ImageJ* software.

### Detection of protein expression

Cells were cultured in 100 mm dishes (2.2 × 10^6^/dish). Treated with drugs for 48 h, cells were collected and washed twice with cold PBS, and then lysed in RIPA and centrifuged to collect the supernatant. The protein concentration was measured by a BCA assay kit. 40–50 μg protein was separated on 8–12% SDS-PAGE and transferred onto a polyvinylidene fluoride membrane (Invitrogen, Carlsbad, CA, USA). After blocking the non-specific site with bovine serum albumin, the membrane was incubated with primary antibody (1,1000 or 1800) at 4 °C overnight, and then incubated for 60 min with a secondary antibody (anti-rabbit IgG, horseradish peroxidase-linked antibody; #7074; Cell Signaling Technologies, Beverly, MA, USA; dilution, 1:2000) at room temperature, respectively. Immunoreactive proteins were detected by HRP-ECL chemiluminescence reagents.

### Statistical analysis

All data were expressed as mean ± SD. Differences between groups were evaluated by the Student’s t test or one way ANOVA followed by Dunnett’s multiple comparison test. *p* < 0.05 was considered as statistically significant.

## Results

### Cardamonin suppressed the glycolysis

**Fig. 1:**
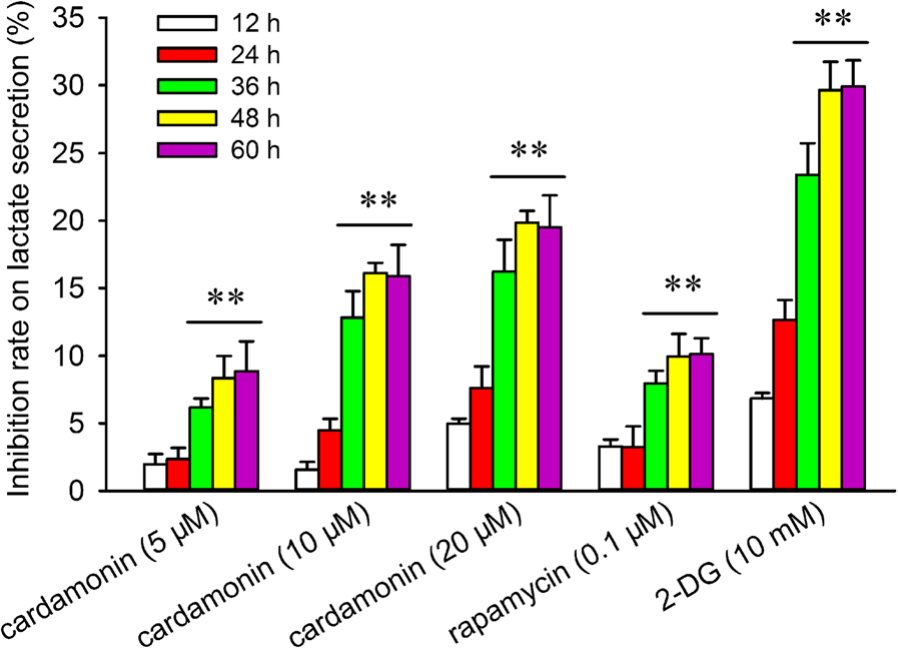
Cardamonin inhibited lactate secretion. SKOV3 cells were treated with cardamonin (5, 10, 20 μM), rapamycin (0.1 μM) and 2-DG (10 mM) for 24, 36, 48, 60 and 72 h. Lactate secretion was analyzed by an assay kit. Inhibition Rate = (1 - drug group/control × 100%). ^**^p < 0.01 vs. 24 h. Data were represented as mean ± SD, *n* = 6

Glycolysis is characterized with the conversion of glucose into lactate [20]. We measured the content of lactate to assess the inhibitory effect of cardamonin on glycolysis. As shown in Fig. 1, the lactate secretion was significantly inhibited by cardamonin in a dose- and time-dependent manner by cardamonin in SKOV3 cells. Treated with indicated drugs for 48 h, the inhibitory effect of cardamonin on lactate secretion is almost the strongest. Similarly, the intracellular ATP content was decreased by cardamonin in a dose-dependent manner at 48 h. Interestingly, the activity of HK and LDH was also reduced by cardamonin and rapamycin, while 2-DG had no effect on these kinases (Table 1).

**Table 1:** Cardamonin inhibited ATP production and the activity of HK and LDH

	ATP (μmoL/g prot)	HK (U/g prot)	LDH (U/g prot)
Control	94.05 ± 0.94	43.63 ± 3.82	307.28 ± 6.71
Cardamonin (5 μM)	87.69 ± 0.79^**^	35.58 ± 3.34^**^	292.94 ± 5.56^**^
Cardamonin (10 μM)	50.98 ± 1.67^**^	33.95 ± 3.04^**^	276.62 ± 4.04^**^
Cardamonin (20 μM)	29.87 ± 1.31^**^	27.46 ± 3.60^**^	225.58 ± 7.50^**^
Rapamycin (0.1 μM)	56.07 ± 1.47^**^	34.14 ± 4.90^**^	286.46 ± 8.68^**^
2-DG (10 mM)	24.33 ± 1.90^**^	43.72 ± 3.12	302.43 ± 4.20

SKOV3 cells were treated with cardamonin (5, 10, 20 μM), rapamycin (0.1 μM) and 2-DG (10 mM) for 48 h, respectively. ATP content and the activity of HK and LDH were analyzed by the assay kits, respectively. ^**^p < 0.01 compared to control. Data were represented as mean ± SD, *n* = 6

*2-DG* 2-deoxy-D-glucose, *HK* hexokinase, *LDH* lactate dehydrogenase

### Cardamonin induced cell autophagy

**Fig. 2:**
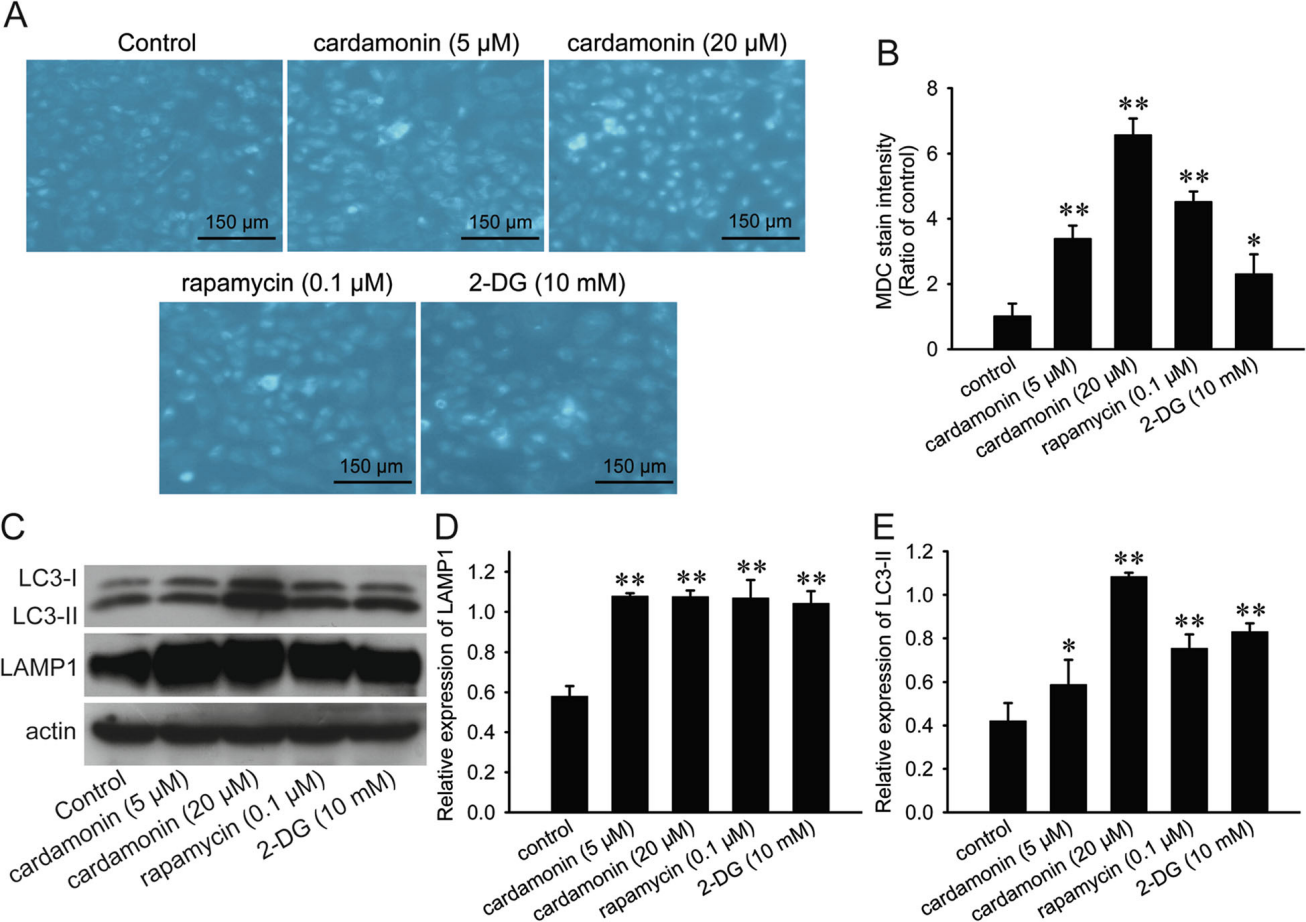
Cardamonin induced cell autophagy. SKOV3 cells were treated with cardamonin (5, 20 μM), rapamycin (0.1 μM) and 2-DG (10 mM) for 48 h, respectively. **a** MDC stain was applied to detect the level of autophagy. **b** The quantitative results of fluorescent intensity. **c** Expression of LC3 and LAMP1 was detected by Western blot. **d**, **e** Quantification of LC3-II and LAMP1 by densitometry of Western blot performed above. ^*^p < 0.05, ^**^p < 0.01 vs. control. Data were represented as mean ± SD, *n* = 3

The autophagy level was confirmed by MDC staining and LC3-II detection in SKOV3 cells. Treated with cardamonin and rapamycin, the fluorescent intensity of MDC stain was significantly elevated (Fig. 2a and b); and the protein level of LC3-II and LAMP1 was increased. As a glycolysis inhibitor, 2-DG also induced autophagy (Fig. 2c-e).

### Cardamonin inhibited the expressions of p-S6K1, p-mTOR, HK2 and increased p-AMPK

**Fig. 3:**
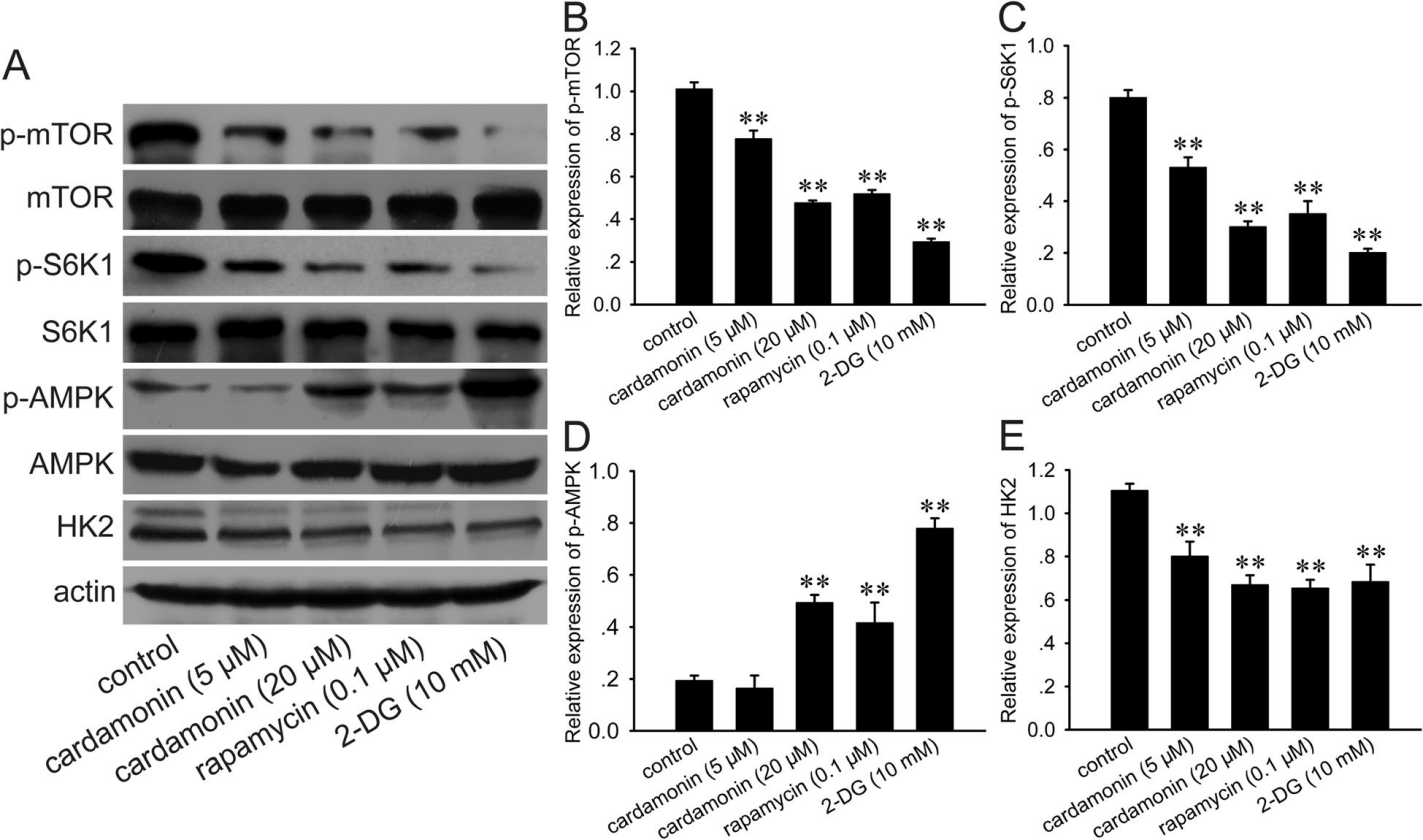
Expression and phosphorylation of S6K1, mTOR, AMPK and HK2. SKOV3 cells were treated with cardamonin (5, 20 μM), rapamycin (0.1 μM) and 2-DG (10 mM) for 48 h, respectively. **a** Expression of protein was detected by Western blot. **b***-***e** Quantification of protein by densitometry of Western blot performed above. ^*^p < 0.05, ^**^p < 0.01 vs. control. Data were represented as mean ± SD, *n* = 3

Since the antitumor activity of cardamonin is correlates with mTOR, the activity of mTOR signaling is investigated. As expected, cardamonin inhibited phosphorylation of mTOR (Ser2448) and its downstream target S6K1 (Thr389). As an energy sensor, AMPK is activated by decreased ATP production. The expression of p-AMPK and HK2 was markedly increased when treated with cardamonin (20 μM), rapamycin or 2-DG for 48 h, respectively. This result was in accordance with the glycolysis inhibition described above (Fig. 3).

### The activity of AMPK was associated with autophagy induction by cardamonin

**Fig. 4:**
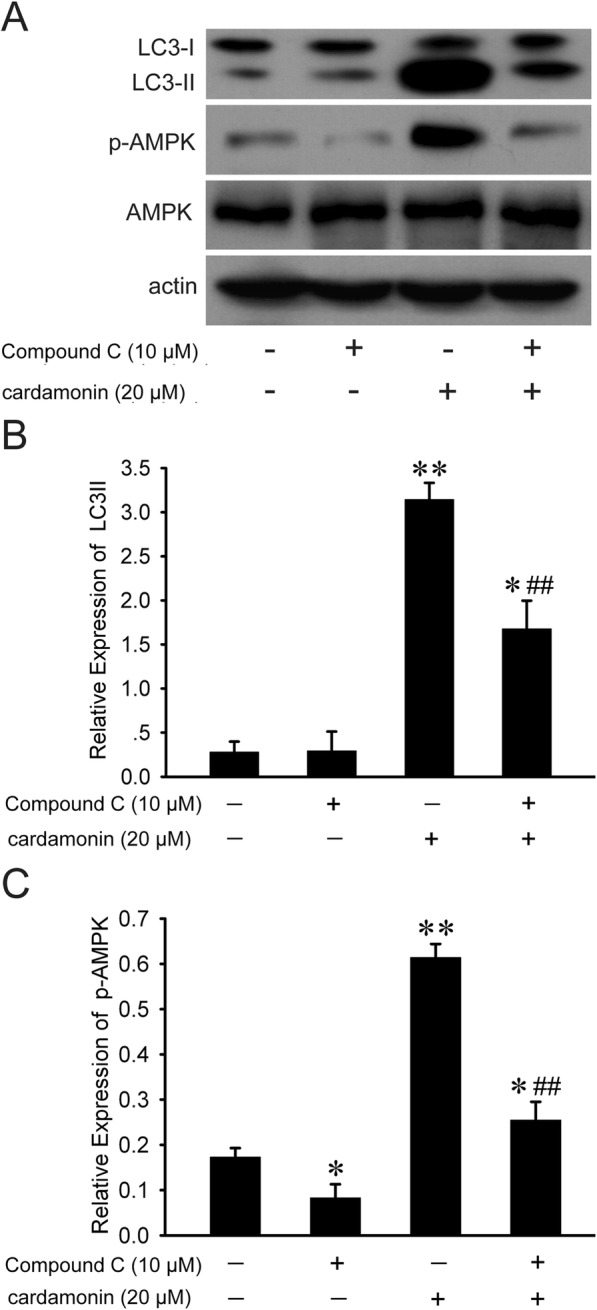
The relationship between the activity of AMPK and autophagy by cardamonin. SKOV3 cells were treated with Compound C (10 μM), cardamonin (20 μM), Compound C (10 μM) + cardamonin (20 μM) for 48 h, respectively. **a** Expression of protein was detected by Western blot. **b**, **c** Quantification of protein by densitometry of Western blot performed above. ^*^p < 0.05, ^**^p < 0.01 vs. control, ^##^*p* < 0.01 vs. cardamonin. Data were represented as mean ± SD, *n* = 3

Next we investigated whether cardamonin-induced autophagy was correlated with glycolysis inhibition. The phosphorylation of AMPK was increased by cardamonin, which was significantly inhibited when combined with the AMPK inhibitor, Compound C. Similarly, the cardamonin-induced expression of LC3-II was reduced by Compound C (Fig. 4).

## Discussion

Autophagy attempts to maintain/restore metabolic homeostasis through digesting the damaged or aged organelles. Recently, Muilenburg et al. have shown that autophagy is a potential strategy for ovarian cancer therapy [21]. Dasatinib can induce cell death through autophagy in ovarian cancer [22]. In addition, increased expression of autophagy protein PEA-15prolonged the life of ovarian cancer patients [23]. Therefore, autophagy provides new ideas for the treatment of ovarian cancer.

LC3 is essential for the elongation of autophagosome membrane. LC3-I conjugates phosphatidylethanolamine to form LC3-II, and LC3-II presents on the outer and inner surfaces of the autophagosome. LAMP1 is a structural protein of the lysosome membrane, which is also considered as an indicator for autophagy [24]. In our present study, the expression of LC3-II and LAMP1 was increased by cardamonin, as well as the acidic autophagy vacuoles stained by MDC. These effects of cardamonin were consistent with that of other similar flavonoids, such as genistein and baicalein [25, 26]. Also, the glycolysis inhibitor 2-DG and mTOR inhibitor rapamycin increased autophagy in SKOV3 cells in the present study. The mechanism of rapamycin and 2-DG on autophagy induction has been demonstrated in other studies. Rapamycin specifically inhibits mTORC1 while that of 2-DG is related to glycometabolism inhibition [27, 28].

When nutrients are deprived, autophagy is enabled by impaired cellular ATP production. As the key point of energy metabolism, glycometabolism plays a unique role in maintaining ovarian cancer cell growth [29–31]. HK is involved in the glycolysis pathway in cancer cells [32]. HK catalyzes the first step of glycolysis. It phosphorylates glucose to glucose-6-phosphate. HK2 is a predominant isoform of HK, which is upregulated in ovarian cancer [33]. It demonstrated that 20(S)-Rg3 inhibited the Warburg effect by targeting STAT3/HK2 pathway in ovarian cancer cells [34]. LDH sustains the glycolysis through catalyzing the regeneration of nicotinamide adenine dinucleotide from reduced nicotinamide adenine dinucleotide in cancer cells [35]. It demonstrates that various flavonoids, including luteolin, wogonin, etc. can inhibit glycolysis [36, 37]. In our study, cardamonin inhibited the activity of HK2 and LDH. It contributes to the glycolysis inhibitory effect of cardamonin. The results were similar with 2-DG, a glucose analog that competitively phosphorylated by HK with glucose and further inhibits glycolysis [28, 38]. It indicated that cardamonin inhibits glycolysis induced energy production. Interestingly, we found that both cardamonin and 2-DG decreased the relative protein expression of HK2. However, only cardamonin but not 2-DG inhibited the activity of HK. The underling mechanism needs to be further illustrated.

mTOR regulates cell growth and metabolism. There exists two different mTOR complexes called mTOR complex 1 (mTORC1) and mTORC2 [39]. The activity of glycolytic enzyme is elevated in an mTORC1-dependent manner in the TSC-deficient cells. Activated mTOR stimulates HIF-1α and coordinately induces the expression of several enzymes for glucose metabolism, including glucose transporters, HK and LDH [32, 40]. It demonstrates that mTORC1 inactivation by rapamycin is critical for glucose metabolism [41, 42]. Rapamycin binds to the FK506-binding protein of 12 kDa (FKBP12) and inhibits the activity of mTORC1 [43]. Our previous study showed that cardamonin had a similar effect with rapamycin on mTOR and its downstream substrates, but this effect was not dependent on FKBP12 [17]. Here, we also observed that cardamonin decreased the phosphorylation of mTOR and S6K1. Therefore, it suggested that the inhibition of glycolysis by cardamonin was regulated by mTORC1 suppression.

AMPK plays an important role in adaptive responses to reduced energy production. In response to nutrition deficiency, accumulation of AMP binding to AMPKγ subunit leads to activation of AMPK [44, 45]. In our present result, cardamonin activated AMPK, which was similar with 2-DG. In order to clarify the activation of AMPK partly results in autophagy induction, we added the Compound C in the experiment. Cardamonin-induced autophagy was reduced by Compound C. All these findings suggested that autophagy stimulation by cardamonin was involved in glycolysis inhibition mediated AMPK activation.

## Conclusion

Cardamonin exhibits antiproliferation activity in various cancer cells. This study also demonstrates that cardamonin-induced autophagy is associated with glycolysis inhibition via mTOR inhibition in SKOV3 cells. Our increased understanding about the mechanism of antitumor effect of cardamonin provides important evidences for further development of cardamonin as a novel therapeutic drug for ovarian cancer.

## Abbreviations

2-DG: 2-deoxy-D-glucose
AMPK: AMP-activated protein kinase
HK2: Hexokinase 2
LAMP1: Lysosome associated membrane protein 1
LC3-II: Microtubule-associated protein 1 Light Chain 3-II
LDH: Lactate dehydrogenase
MDC: Monodansylcadaverin
mTOR: Mammalian target of rapamycin
mTORC1: Mammalian target of rapamycin complex 1
S6K1: Ribosome S6 kinase 1
